# Generation of Isogenic iPSC Lines for Studying the Effect of the p.N515del (c.1543_1545delAAC) Variant on MYBPC3 Function and Hypertrophic Cardiomyopathy Pathogenesis

**DOI:** 10.3390/ijms252312900

**Published:** 2024-11-30

**Authors:** Sophia V. Pavlova, Angelina E. Shulgina, Julia M. Minina, Suren M. Zakian, Elena V. Dementyeva

**Affiliations:** 1 Federal Research Centre Institute of Cytology and Genetics, Siberian Branch of the Russian Academy of Sciences, 630090 Novosibirsk, Russia; spav@bionet.nsc.ru (S.V.P.); SulginaAE@bionet.nsc.ru (A.E.S.); minina_jul@mail.ru (J.M.M.); zakian@bionet.nsc.ru (S.M.Z.); 2 Institute of Chemical Biology and Fundamental Medicine, Siberian Branch of the Russian Academy of Sciences, 630090 Novosibirsk, Russia

**Keywords:** hypertrophic cardiomyopathy, variants of unknown significance, induced pluripotent stem cells, CRISPR/Cas9, iPSC-derived cardiomyocytes

## Abstract

The clinical significance of numerous cardiovascular gene variants remains to be determined. CRISPR/Cas9 allows for the introduction and/or correction of a certain variant in induced pluripotent stem cells (iPSCs). The resulting isogenic iPSC lines can be differentiated into cardiomyocytes and used as a platform to assess the pathogenicity of the variant. In this study, isogenic iPSC lines were generated for a variant of unknown significance found previously in a patient with hypertrophic cardiomyopathy (HCM), p.N515del (c.1543_1545delAAC) in *MYBPC3*. The deletion was corrected with CRISPR/Cas9 in the patient-specific iPSCs. The iPSC lines with the corrected deletion in *MYBPC3* maintained pluripotency and a normal karyotype and showed no off-target CRISPR/Cas9 activity. The isogenic iPSC lines, together with isogenic iPSC lines generated earlier via introducing the p.N515del (c.1543_1545delAAC) variant in *MYBPC3* of iPSCs of a healthy donor, were differentiated into cardiomyocytes. The cardiomyocytes derived from both panels of the isogenic iPSCs showed an increased size in the presence of the deletion in *MYBPC3*, which is one of the HCM traits at the cellular level. This finding indicates the effectiveness of these iPSC lines for studying the impact of the variant on HCM development.

## 1. Introduction

Hypertrophic cardiomyopathy (HCM) is the most frequent cardiomyopathy with an autosomal dominant inheritance pattern and an overall prevalence of 0.2% [1]. The disease is characterized by abnormal thickening of the left ventricle walls and can be accompanied by hypertrophy of the interventricular septum, left ventricular outflow tract obstruction, diastolic dysfunction, progressive heart failure, and atrial or ventricular arrhythmias. Annual mortality is estimated to be 5–6% [2]. HCM was found to be inherited in approximately 60% of cases. The genetics underlying the disease are quite complicated. Variants causing HCM were revealed in genes encoding sarcomere and sarcomere-associated proteins and those regulating calcium homeostasis. Moreover, HCM is one of the clinical manifestations caused by variants in genes associated with several metabolic disorders, neuromuscular diseases, and RASopathies [3,4]. Nevertheless, 40–50% of inherited HCM cases are due to variants in *MYBPC3*, which encodes a sarcomeric protein, myosin-binding protein C (cMyBP-C). The protein interacts with actin, myosin, and titin, thereby maintaining sarcomeric structure and regulating cardiomyocyte contraction and relaxation [5]. Most pathogenic variants in *MYBPC3* are truncating and result in premature termination codons via nonsense and frameshift variants or splice site alternations, which causes synthesis of a truncated mRNA transcript and protein from the allele [6,7]. Pathogenetic mechanisms triggered by the truncating variants are supposed to be allele instability and haploinsufficiency because of nonsense-mediated RNA decay and the effects of the variants on protein stability [6,8,9]. However, approximately 15% of *MYBPC3*-associated HCM cases are accounted for by nontruncating variants that include missense variants and in-frame deletions and insertions [6]. Whereas the truncating variants are evenly distributed along *MYBPC3*, the nontruncating ones are concentrated in three immunoglobulin-like domains (C3, C6, and C10) of the protein [10]. The impact of the nontruncating variants on MYBPC3 structure and function as well as HCM pathogenesis is much less understood and needs to be elucidated.

A heterozygous p.N515del (c.1543_1545delAAC) variant was identified in the C3 domain of *MYBPC3* during genetic analysis of HCM patients [11] (rs730880643, MIM *600958 for MYBPC3, MIM #115197 for *MYBPC3*-associated HCM). The non-frameshift trinucleotide deletion is a rare variant with minor allele frequency being 0.00000186 (gnomAD v4.1.0, https://gnomad.broadinstitute.org/, accessed on 29 October 2024). It was also found in a number of genetic screenings of patients with HCM and dilated cardiomyopathy [12,13,14,15,16]. However, interpretation of the variant clinical significance is challenging, as multiple in silico tools fail to predict pathogenicity of deletions. According to the ClinVar database (https://www.ncbi.nlm.nih.gov/clinvar/, accessed on 29 October 2024), the clinical significance of this nontruncating variant is currently uncertain. The lack of in silico data makes using other approaches to assess pathogenicity of the variant much more relevant.

CRISPR/Cas9 opens new prospects in modeling and studying inherited cardiovascular diseases [17,18,19,20]. One of the CRISPR/Cas9 applications is establishing contribution of variants of unknown significance to the development of cardiovascular diseases [21]. The approach has been successfully used to find out the pathogenetic contribution of several variants to HCM development and expressivity [22,23,24,25]. The technology is based on using CRISPR/Cas9 for introducing a variant of interest in induced pluripotent stem cells (iPSCs) of healthy donor and/or its correction in patient-specific iPSCs. The resulting isogenic iPSC lines are characterized by the presence/absence of the variant and share a genetic background. Comparing the properties of cardiomyocytes derived from the isogenic iPSC lines allows for establishing a correlation between the presence of the variant and manifestations of pathological phenotype and making conclusions about the clinical significance of the genetic variant.

We previously used CRISPR/Cas9 to introduce the p.N515del (c.1543_1545delAAC) variant in *MYBPC3* of iPSCs from a healthy donor [26]. In the study, we created the second panel of isogenic iPSC lines by correction of the deletion in *MYBPC3* of patient-specific iPSCs with CRISPR/Cas9. Cardiomyocytes derived from all the isogenic iPSC lines demonstrated an increased size only in the presence of the p.N515del (c.1543_1545delAAC) variant in *MYBPC3*. This indicates potential pathogenicity of the variant in *MYBPC3* and the effectiveness of the isogenic iPSC lines for studying the impact of the variant on MYBPC3 function and HCM development.

## 2. Results

### 2.1. Correction of p.N515del (c.1543_1545delAAC) Variant in MYBPC3 of Patient-Specific iPSCs

**Figure 1:**
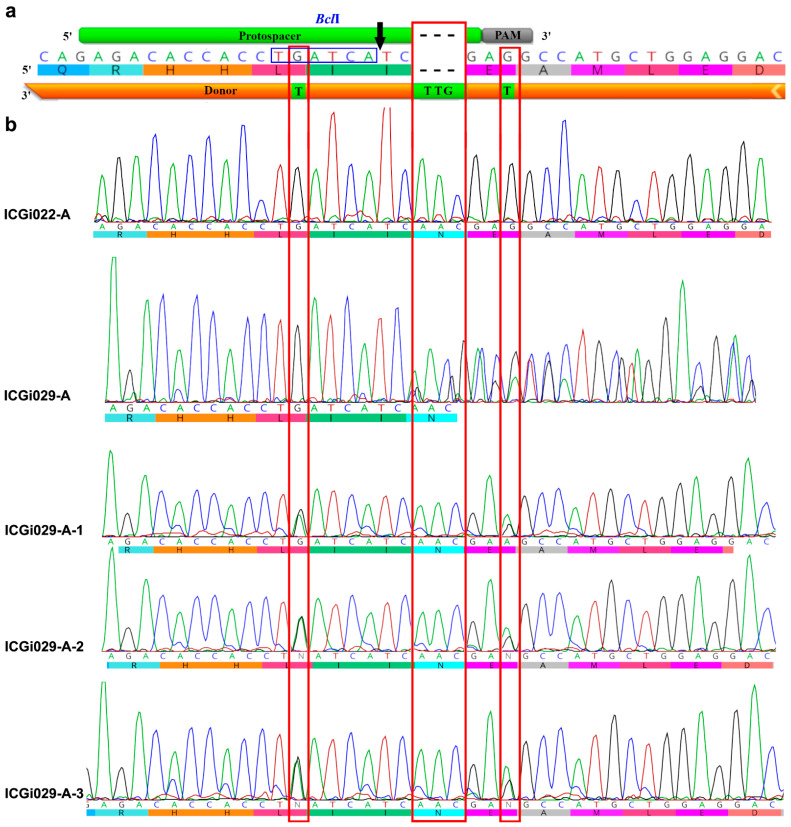
Correction of *MYBPC3* in the patient-specific iPSC line. (**a**) Location of protospacer for single guide RNA (in green), PAM (in grey), and single-stranded donor oligonucleotide (in orange) in *MYBPC3* exon 17. The point of cleavage with CRISPR/Cas9 is marked with a black arrow. A site for the *Bcl*I restriction endonuclease is indicated with blue rectangle. (**b**) An example of three iPSC clones with the corrected p.N515del (c.1543_1545delAAC) variant in *MYBPC3* (ICGi029-A-1, ICGi029-A-2, ICGi029-A-3). Nucleotide sequences of the same region in the patient-specific iPSCs (ICGi029-A) and iPSCs of the healthy donor (ICGi022-A) are shown for comparison. Positions of the target trinucleotide deletion and two synonymous substitutions in PAM and the *Bcl*I restriction site are indicated with red rectangles.

To correct the p.N515del (c.1543_1545delAAC) variant in *MYBPC3* exon 17 of patient-specific iPSCs, introducing a double-strand break with CRISPR/Cas9 and homology-directed repair with single-stranded donor oligonucleotide were used. We chose a protospacer for single-guide RNA and Protospacer Ajacent Motif (PAM) taking into account two points. The first point is that the double-strand break should be located as close as possible to the deletion – in 2 bp for *MYBPC3* correction (Figure 1a). The second one is an opportunity to make a synonymous substitution in PAM to exclude repetitive *MYBPC3* editing with CRISPR/Cas9. Benchling and IDT (https://www.benchling.com/ and https://www.idtdna.com/, accessed on 13 May 2022) showed low probability of off-target activity of the selected CRISPR/Cas9. The protospacer also spanned the trinucleotide deletion so that CRISPR/Cas9 was able to make double-strand breaks only in the mutant allele (Figure 1a). The single-stranded donor oligonucleotide was designed to correspond to wild-type nucleotide sequence of *MYBPC3* exon 17 but contained two synonymous substitutions. The first substitution changed PAM. The second one disrupted a site for the *Bcl*I restriction endonuclease, which creates an additional possibility to reveal the deletion correction using restriction analysis (Figure 1a). The nucleotide sequences of the protospacer and single-stranded donor oligonucleotide can be found in Table S1. CRISPR/Cas9 delivery in ribonucleoprotein (RNP) complexes was observed to cause a higher rate of editing events and reduced rate of off-target cleavage compared to plasmid DNA transfection [27]. Therefore, the approach was selected for CRISPR/Cas9 delivery in this study. To increase the stability of CRISPR/Cas9 RNP complexes and efficiency of the editing process, the single-guide RNA and single-stranded donor oligonucleotide were protected with additional chemical modifications [28]: 2′-O-methyl 3′ phosphorothioate and 3′ phosphorothioate, respectively, at the first three 5′ and 3′ terminal nucleotides.

CRISPR/Cas9 RNP complexes, together with the single-stranded donor oligonucleotide, were electroporated into iPSCs of the ICGi029-A line derived from a carrier of the p.N515del (c.1543_1545delAAC) variant in *MYBPC3* [29]. After subcloning of the electroporated iPSCs, 99 iPSC clones have been generated. The iPSC clones were analyzed using Sanger sequencing of *MYBPC3* exon 17, which was found to be more informative for understanding editing events than restriction analysis. Homology-directed repair, which resulted in the deletion correction and restoration of the wild-type nucleotide sequence, was observed in 10 (10.1%) of the iPSC clones (Figure 1b). The other iPSC clones contained indels due to non-homologous end joining or were not edited with CRISPR/Cas9. Three iPSC clones with the corrected p.N515del (c.1543_1545delAAC) variant in *MYBPC3* (ICGi029-A-1, ICGi029-A-2, ICGi029-A-3) were checked for CRISPR/Cas9 off-target activity. Comparison of nucleotide sequences of five most probable sites of CRISPR/Cas9 off-target cleavage and their surroundings in the iPSC lines with the corrected deletion and the ICGi029-A line used for MYBPC3 editing demonstrated the absence of CRISPR/Cas9 off-target activity (Figure S1a).

### 2.2. iPSC Lines with the Corrected Deletion in MYBPC3 Maintain Pluripotency and Normal Karyotype

**Figure 2:**
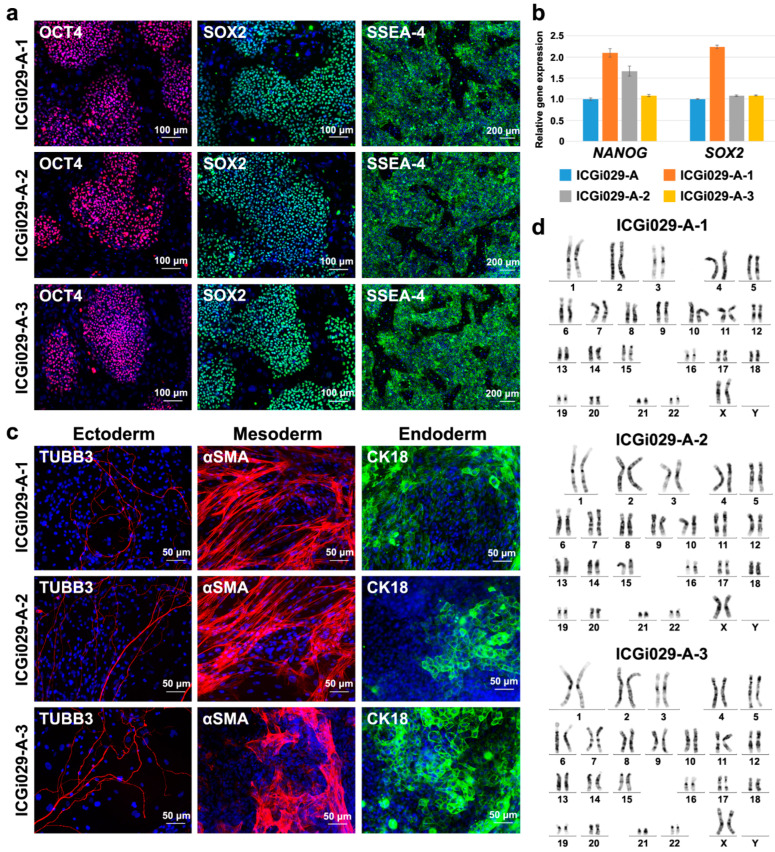
The iPSC lines with corrected p.N515del (c.1543_1545delAAC) variant in *MYBPC3* (ICGi029-A-1, ICGi029-A-2, ICGi029-A-3) retain the pluripotent state and normal karyotype. (**a**) The iPSC lines with the corrected deletion express the OCT4 and SOX2 transcription factors and SSEA-4 surface antigen. Scale bar—100 µm and 200 µm (for SSEA-4). (**b**) Pluripotency genes, *NANOG* and *SOX2*, have similar expression levels in the iPSC lines with corrected p.N515del (c.1543_1545delAAC) variant in *MYBPC3* and the patient-specific ICGi029-A iPSC line used for *MYBPC3* correction. The data are presented as mean ± SEM. (**c**) The iPSC lines corrected with CRISPR/Cas9 give rise to derivatives of ectoderm (βIII-tubulin, TUBB3), mesoderm (smooth muscle α-actin, αSMA), and endoderm (cytokeratin 18, CK18) during spontaneous differentiation in embryoid bodies. Scale bar—50 µm. (**d**) The iPSC lines with corrected p.N515del (c.1543_1545delAAC) variant in *MYBPC3* retain normal karyotype, 46,XX.

To use the iPSC lines with the corrected p.N515del (c.1543_1545delAAC) variant in *MYBPC3* in further studies, it was necessary to confirm retention of their pluripotent status and normal karyotype. The three iPSC lines corrected with CRISPR/Cas9 (ICGi029-A-1, ICGi029-A-2, ICGi029-A-3) were shown to express transcription factors (OCT4 and SOX2) and surface antigens (SSEA-4) characteristic of the pluripotent state (Figure 2a). In addition, the expression levels of two genes involved in pluripotency maintenance (*NANOG* and *SOX2*) were comparable between the iPSC lines with the corrected deletion in *MYBPC3* and the ICGi029-A line used for *MYBPC3* editing (Figure 2b). Another property of pluripotent stem cells is a capacity to be differentiated into derivatives of three germ layers. We performed spontaneous differentiation of the three iPSC lines with the corrected p.N515del (c.1543_1545delAAC) variant in *MYBPC3* in embryoid bodies and revealed cells expressed markers of three germ layers among differentiated derivatives using immunofluorescence staining (Figure 2c). Karyotype analysis showed that the iPSC lines corrected with CRISPR/Cas9 possessed the normal number and structure of chromosomes (46,XX, Figure 2d). The iPSC lines with the corrected p.N515del (c.1543_1545delAAC) variant in *MYBPC3* were also negative for mycoplasma contamination (Figure S1b). STR analysis confirmed identity of the iPSC lines with the corrected deletion in *MYBPC3* to the original patient-specific iPSC line.

Thus, despite *MYBPC3* editing, the iPSC lines corrected with CRISPR/Cas9 retained pluripotent status and the normal karyotype and can be used for studying pathogenetic contribution of the p.N515del (c.1543_1545delAAC) variant in *MYBPC3* to the HCM development.

### 2.3. Presence of p.N515del (c.1543_1545delAAC) Variant in MYBPC3 Correlates with Increased Size of Cardiomyocytes Derived from Isogenic iPSC Lines

The patient-specific ICGi029-A iPSC line and three iPSC lines with the corrected p.N515del (c.1543_1545delAAC) variant in *MYBPC3* (ICGi029-A-1, ICGi029-A-2, ICGi029-A-3), together with the other panel of isogenic iPSC lines comprised one iPSC lines from the healthy donor (the ICGi022-A line, [30]) and three iPSC lines with the introduced deletion in *MYBPC3* (ICGi022-A-3, ICGi022-A-4, ICGi022-A-5) [26], were directly differentiated into cardiomyocytes. The iPSC-derived cardiomyocytes were generated using temporal modulation of the Wnt signaling pathway and a medium lacking insulin during the first 7 days of cardiac differentiation [31,32].

**Figure 3:**
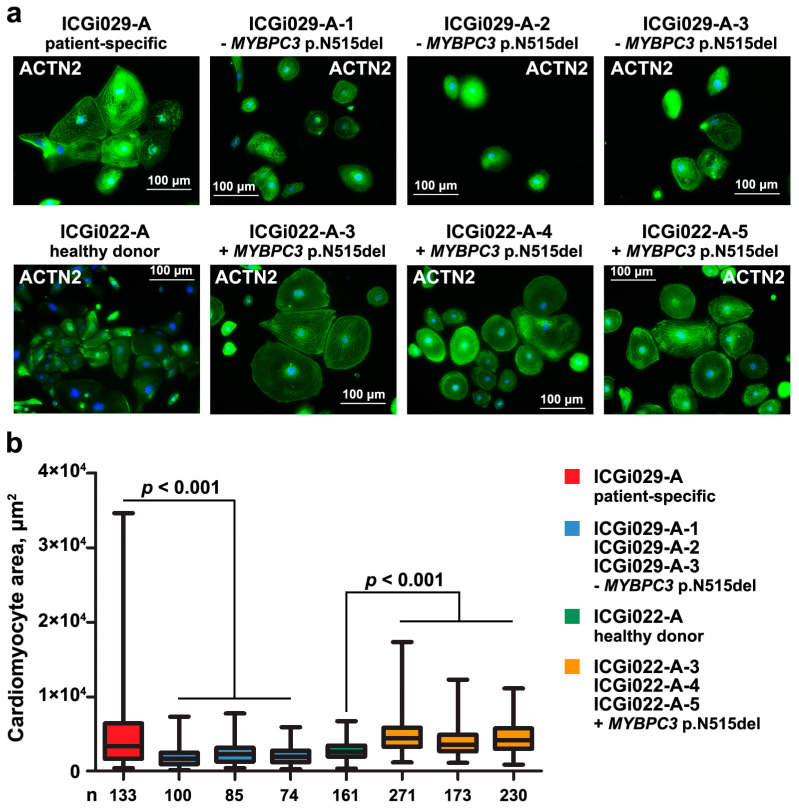
The presence of the p.N515del (c.1543_1545delAAC) variant in *MYBPC3* correlates with an increased size of cardiomyocytes derived from isogenic iPSC lines generated by correction and introducing of the deletion with CRISPR/Cas9. (**a**) Detecting boundaries of cardiomyocytes derived from the patient-specific iPSC line (ICGi029-A), three iPSC lines with the corrected p.N515del (c.1543_1545delAAC) variant in *MYBPC3* (ICGi029-A-1, ICGi029-A-2, ICGi029-A-3), the iPSC line from the healthy donor (ICGi022-A), and three iPSC lines with the introduced deletion in *MYBPC3* (ICGi022-A-3, ICGi022-A-4, ICGi022-A-5). ACTN2, alpha-actinin-2. Scale bar—100 µm. (**b**) Results of measuring areas of cardiomyocytes derived from the patient-specific iPSC line (ICGi029-A), three iPSC lines with corrected p.N515del (c.1543_1545delAAC) variant in *MYBPC3* (ICGi029-A-1, ICGi029-A-2, ICGi029-A-3), the iPSC line from the healthy donor (ICGi022-A), and three iPSC lines with the introduced deletion in *MYBPC3* (ICGi022-A-3, ICGi022-A-4, ICGi022-A-5). The data are presented as mean with minimal and maximal values. Number of analyzed cardiomyocytes (n) is provided for each iPSC line.

To confirm a capacity of two panels of the isogenic iPSC lines to be useful for studying the pathogenicity of the p.N515del (c.1543_1545delAAC) variant in *MYBPC3*, we compared areas of the iPSC-derived cardiomyocytes. The cardiomyocyte boundaries were detected using immunofluorescence staining with antibodies to a cardiomyocyte-specific marker, alpha-actinin-2 (Figure 3a). The calculation of the cardiomyocyte areas demonstrated that the presence of the deletion in *MYBPC3* correlated with an increased size of the iPSC-derived cardiomyocytes (Figure 3b). The correction of the p.N515del (c.1543_1545delAAC) variant in *MYBPC3* led to a decreased size of iPSC-derived cardiomyocytes in comparison with patient-specific ones, whereas introducing the deletion resulted in an increased size of iPSC-derived cardiomyocytes compared to those from the healthy donor.

## 3. Discussion

*MYBPC3* is one of the two genes where HCM-causing variants are the most frequent [3,4]. According to the ClinVar database (https://www.ncbi.nlm.nih.gov/clinvar/, accessed on 29 October 2024), 4203 variants in the gene have been described. About 39.19% (1647 out of 4203) of the variants have uncertain significance and 7.4% (311 out of 4203) of the variants are characterized by conflicting classifications of pathogenicity. To date variant classification is based mainly on minor allele frequency in the general population, data from patients’ genetic screenings, and multiple in silico tools of prediction for pathogenicity. However, the in silico methods are effective only for single nucleotide substitutions and cannot predict pathogenicity of other types of variants, e.g., deletions and insertions.

In a genetic screening of HCM patients, we found a deletion of uncertain significance in *MYBPC3*, p.N515del (c.1543_1545delAAC) [11]. The variant is located in the immunoglobulin-like C3 domain whereas the MYBPC3 sites interacting with actin, myosin, and titin are situated at the N- and C-termini of the protein (the C0-C2 and C8-C10 domains) [5]. Thus, the deletion does not disrupt the binding sites of the sarcomeric proteins. An analysis of the ClinVar database (https://www.ncbi.nlm.nih.gov/clinvar/, accessed on 21 November 2024) showed 59 pathogenic and likely pathogenic variants in the *MYBPC3* C3 domain. 25 (42.37%) of the variants were deletions, almost all of which interfered with reading frame. Only one deletion, p.K505del (c.1513_1515delAAG, rs727504287), was non-frameshift and caused loss of an amino acid, as is the p.N515del (c.1543_1545delAAC) variant. This variant is classified as likely pathogenic and was found in several genetic studies of HCM patients [33,34,35,36,37,38]. The pathogenicity of the p.K505del (c.1513_1515delAAG) variant implies that even single-amino-acid deletions in the C3 domain may have an impact on MYBPC3 function and HCM development. A possible explanation may come from two functional studies of another pathogenic variant in the C3 domain, p.E542Q (c.1624G>C, rs121909374). The variant was found to shift the balance from thin filament interaction toward myosin binding [39] and to diminish significantly super-relaxed state of myosin [40]. Thus, the variants in the C3 domain are likely to influence the kinetics of cardiomyocyte contraction. We also tried to analyze 3D structure of the C3 domain around p.N515 using RCSB PDB Mol* 3D Viewer (https://www.rcsb.org/3d-view, accessed on 22 November 2024) (Figure S2). It is very tempting to assume that the p.N515 deletion disrupts two hydrogen bonds between neighboring p.A517 and two other amino acids from the C3 domain, p.Q469 and p.G468, which may affect the domain structure and function. However, all the data are quite speculative and functional studies of the p.N515del (c.1543_1545delAAC) variant are required.

The absence of an in silico prediction for pathogenicity and data on functional analysis prompted us to generate isogenic iPSC lines with CRISPR/Cas9 to find out an impact of the non-frameshift trinucleotide deletion on MYBPC3 function and HCM pathogenesis. In our previous study, the p.N515del (c.1543_1545delAAC) variant was introduced in *MYBPC3* of iPSCs from the healthy donor. Three iPSC lines were generated and characterized in detail [26]. However, all the iPSC lines were homozygous at the deletion. Therefore, the panel of isogenic iPSC lines may not reproduce exactly the situation in the HCM patient who was heterozygous at the p.N515del (c.1543_1545delAAC) variant. The aim of this study was to correct the deletion in *MYBPC3* of patient-specific iPSCs. The specificity of the selected CRISPR/Cas9 to the mutant allele increased the probability of generating isogenic control for the patient-specific iPSC line. The variant correction gives us an opportunity to examine the consequences of the deletion in a heterozygous state and under two different genetic backgrounds, which will allow us to make more solid conclusions about the variant pathogenicity. As a result, three iPSC lines with the corrected p.N515del (c.1543_1545delAAC) variant in *MYBPC3* were generated. The retention of the pluripotent properties and normal karyotype was confirmed.

We further validated a capacity of the two panels of isogenic iPSC lines to be a platform for studying the contribution of the p.N515del (c.1543_1545delAAC) variant in *MYBPC3* to HCM pathogenesis. An increase in size, which is one of the HCM traits at the cellular level [41,42,43,44,45,46], was assessed in cardiomyocytes derived from the isogenic iPSC lines. The cardiomyocytes that carried the deletion in *MYBPC3* were shown to have an increased size compared to their isogenic controls. This indicates the suitability of the isogenic iPSC lines for examining the p.N515del (c.1543_1545delAAC) variant in *MYBPC3*. However, more studies need to be performed using the isogenic iPSC lines to confirm the pathogenicity of the deletion.

## 4. Materials and Methods

### 4.1. MYBPC3 Correction in Patient-Specific iPSCs

CRISPR/Cas9 RNP complexes were formed by incubation of 50 pmol of single-guide RNA (GenScript, Nanjing, China) and 20 pmol of Cas9_NLS (NEB, Ipswich, MA, USA) for 20 min at room temperature. The RNP complexes, together with 300 ng of single-stranded donor oligonucleotide (Biolegio, Nijmegen, The Netherlands), were delivered into 1 × 10^5^ of patient-specific iPSCs (the ICGi029-A line, [29]) by electroporation using Neon Transfection System 10 μL Kit (Thermo Fisher Scientific, Waltham, MA, USA) under the following conditions: 1100 V, 30 ms, one time. Then, 48 h after electroporation, the cells were subcloned. Individual iPSC clones were cultivated on a feeder layer in KnockOut DMEM supplemented with 15% KnockOut Serum Replacement, 0.1 mM MEM Non-Essential Amino Acids Solution, 1× penicillin-streptomycin, 1 mM GlutaMAX (all reagents from Thermo Fisher Scientific, Waltham, MA, USA), 0.05 mM 2-mercaptoethanol (Amresco, Solon, OH, USA), and 10 ng/mL bFGF (SCI-store, Moscow, Russia). TrypLE™ Express Enzyme (Thermo Fisher Scientific, Waltham, MA, USA) was used for iPSC clone passaging.

### 4.2. Search for Editing Events in MYBPC3 and Off-Target Cleavage with CRISPR/Cas9

To reveal *MYBPC3* correction and off-target cleavage with CRISPR/Cas9 in the iPSC clones, PCR products contained a part of *MYBPC3* exon 17 or CRISPR/Cas9 off-target sites predicted with IDT (https://www.idtdna.com/, accessed on 3 April 2023) were analyzed by Sanger sequencing at the SB RAS Genomics Core Facility (Novosibirsk, Russia). PCR was conducted with BioMaster HS-Taq PCR-Color (2×) (Biolabmix, Novosibirsk, Russia) on a T100 Thermal Cycler (Bio-Rad, Hercules, CA, USA) under the following conditions: 95 °C—3 min; 35 cycles: 95 °C—30 s, 58 °C (*MYBPC3*) or 62 °C (off-target sites)—30 s, 72 °C—30–40 s; 72 °C—5 min. The primer nucleotide sequences can be found in Table S1.

### 4.3. Spontaneous Differentiation in Embryoid Bodies

Embryoid bodies were formed by iPSC disaggregation with 0.15% Collagenase IV (Thermo Fisher Scientific, Waltham, MA, USA) and subsequent cultivation of the cell clumps for two weeks on 1% agarose-coated dishes in DMEM/F12 (1:1) medium supplemented with 15% KnockOut Serum Replacement, 0.1 mM MEM Non-Essential Amino Acids Solution, 1× penicillin-streptomycin, 1 mM GlutaMAX (all reagents from Thermo Fisher Scientific, Waltham, MA, USA). For further analysis, the embryoid bodies were seeded for an additional week on Matrigel (Corning, New York, NY, USA)-coated 8-well Chambered Coverglass (Thermo Fisher Scientific, Waltham, MA, USA). The medium was changed every 3 days.

### 4.4. Immunofluorescence Staining

iPSCs and their differentiated derivatives were treated with 4% paraformaldehyde (Sigma–Aldrich, Darmstadt, Germany) for 10 min, 0.4% Triton-X100 (Sigma–Aldrich, Darmstadt, Germany) for 10 min, PBS 2 times for 5 min, 1% bovine serum albumin (VWR, Solon, OH, USA) for 30 min. In case of SSEA-4, iPSC treatment was conducted without the permeabilization step in Triton-X100. Cells were further incubated overnight at 4 °C with primary antibodies, washed twice for 15 min in PBS, incubated for 1 h at room temperature with secondary antibodies, and washed one more time in PBS. The primary and secondary antibodies can be found in Table S1. DAPI (Sigma–Aldrich, Darmstadt, Germany) was used to counterstain nuclei. Immunofluorescence staining was analyzed on a Nikon Eclipse Ti-E with NIS Elements Advanced Research software version 4.30 (Nikon, Tokyo, Japan).

### 4.5. Quantitative Reverse Transcription PCR

TRIzol Reagent and DNA-free™ DNA Removal Kit (all reagents from Thermo Fisher Scientific, Waltham, MA, USA) were used to isolate and purify RNA according to the manufacturers’ instructions. cDNA was synthetized with the M-MuLV reverse transcriptase (Biolabmix, Novosibirsk, Russia). Quantitative reverse transcription PCR was carried out with BioMaster HS-qPCR SYBR Blue 2× (Biolabmix, Novosibirsk, Russia) on a QuantStudio™ 5 Real-Time PCR System (Applied Biosystems, Life Technologies Holdings Pte. Ltd., Singapore). The data were normalized via the ΔΔCT method, using *B2M* as a reference gene. The primer nucleotide sequences can be found in Table S1.

### 4.6. Karyotyping

Detailed description of iPSC plating, treatment with Colcemide and hypotonic solution, as well as metaphase collection and fixation can be found in [47]. Karyotype analysis was carried out using an Axioplan 2 (Zeiss, Oberkochen, Germany) and the ISIS 5 program (MetaSystems, Altlussheim, Germany). No fewer than 50 metaphase plates were analyzed for each iPSC line.

### 4.7. Detection of Mycoplasma Contamination

PCR was used to validate the absence of mycoplasma contaminations in the iPSC lines. PCR was performed as described in Section 4.2 under the following conditions: 95 °C—3 min; 35 cycles: 95 °C—15 s, 67 °C—15 s, 72 °C—20 s; 72 °C—5 min. The primer nucleotide sequences can be found in Table S1.

### 4.8. STR Analysis

STR analysis of the iPSC lines was conducted by the Genoanalytika Company using COrDIS EXPERT 26 Kit (Gordis, Moscow, Russia).

### 4.9. iPSC Differentiation into Cardiomyocytes

To induce iPSC differentiation into the mesoderm, the canonical Wnt pathway was activated by adding for 48 h the RPMI/B27-ins medium with 6 µM CHIR99021 (Sigma–Aldrich, Darmstadt, Germany). To direct the mesodermal differentiation into cardiomyocytes, Wnt was repressed by adding on day 3 for 48 h the RPMI/B27-ins medium with an Wnt inhibitor: 5 µM IWP2 (Sigma–Aldrich, Darmstadt, Germany) or 2 µM Wnt-C59 (Selleck Chemicals, Houston, TX, USA). On day 7, the medium was changed to the RPMI/B27 medium. After the appearance of spontaneously contracting zones (day 9), differentiated cells were cultivated for 7–9 days in a metabolic medium [32] to purify cardiomyocytes from other cell types.

The RPMI/B27-ins medium is RPMI 1640 medium supplemented with 1× penicillin-streptomycin and 1× B27 Supplement minus insulin (all reagents from Thermo Fisher Scientific, Waltham, MA, USA). The RPMI/B27 medium is RPMI 1640 medium supplemented with 1× penicillin-streptomycin and 1× B27 Supplement (all reagents from Thermo Fisher Scientific, Waltham, MA, USA). The metabolic medium is RPMI 1640 medium without D-glucose (Thermo Fisher Scientific, Waltham, MA, USA) supplemented with 1× penicillin-streptomycin, 500 µg/mL bovine serum albumin (VWR, Solon, OH, USA), 213 µg/mL L-ascorbic acid 2-phosphate sesquimagnesium salt hydrate, and 5 mM sodium DL-lactate (all reagents from Sigma–Aldrich, Darmstadt, Germany).

### 4.10. Measuring Cardiomyocyte Size

Purified cardiomyocytes were disaggregated with 0.25% trypsin, plated on Matrigel-coated wells, and cultivated in the RPMI/B27 medium. Cardiomyocyte boundaries were defined using immunofluorescence staining with antibodies to alpha-actinin-2 (Table S1) as described above. Nikon Eclipse Ti-E and NIS Elements Advanced Research software version 4.30 (Nikon, Tokyo, Japan) were used to obtain images of cardiomyocytes in random fields of view. Cell areas were calculated with ImageJ 1.53k. The number of the analyzed cardiomyocytes for the first panel of isogenic iPSC lines (the deletion correction) varied from 74 to 133 and that for the second panel of isogenic iPSC lines (introducing the deletion) varied from 161 to 271.

### 4.11. Statistics

One-way ANOVA with Tukey’s correction for multiple comparisons was used to calculate the statistical significance of differences in size between cardiomyocytes derived from isogenic iPSC lines. The calculations and box plot were performed in GraphPad Prism Version 5.00. *p*-values < 0.05 were considered to be significant.

## Data Availability

The data on characterization of the iPSC lines are available in the Human Pluripotent Stem Cell Registry (https://hpscreg.eu/cell-line/ICGi029-A-1, https://hpscreg.eu/cell-line/ICGi029-A-2, https://hpscreg.eu/cell-line/ICGi029-A-3 accessed on 29 October 2024).

## Supplementary Materials

The following supporting information can be downloaded at: https://www.mdpi.com/article/10.3390/ijms252312900/s1.
