# Supplementary material for: Generation of Isogenic iPSC Lines for Studying the Effect of the p.N515del (c.1543_1545delAAC) Variant on MYBPC3 Function and Hypertrophic Cardiomyopathy Pathogenesis
Source: Int J Mol Sci. 2024 Nov 30;25(23):12900. doi: 10.3390/ijms252312900 (PMC11641397; doi:10.3390/ijms252312900)
Supplement: Supplementary file 1 [file ijms-25-12900-s001.zip › ijms-3321753-supplementary.pdf]

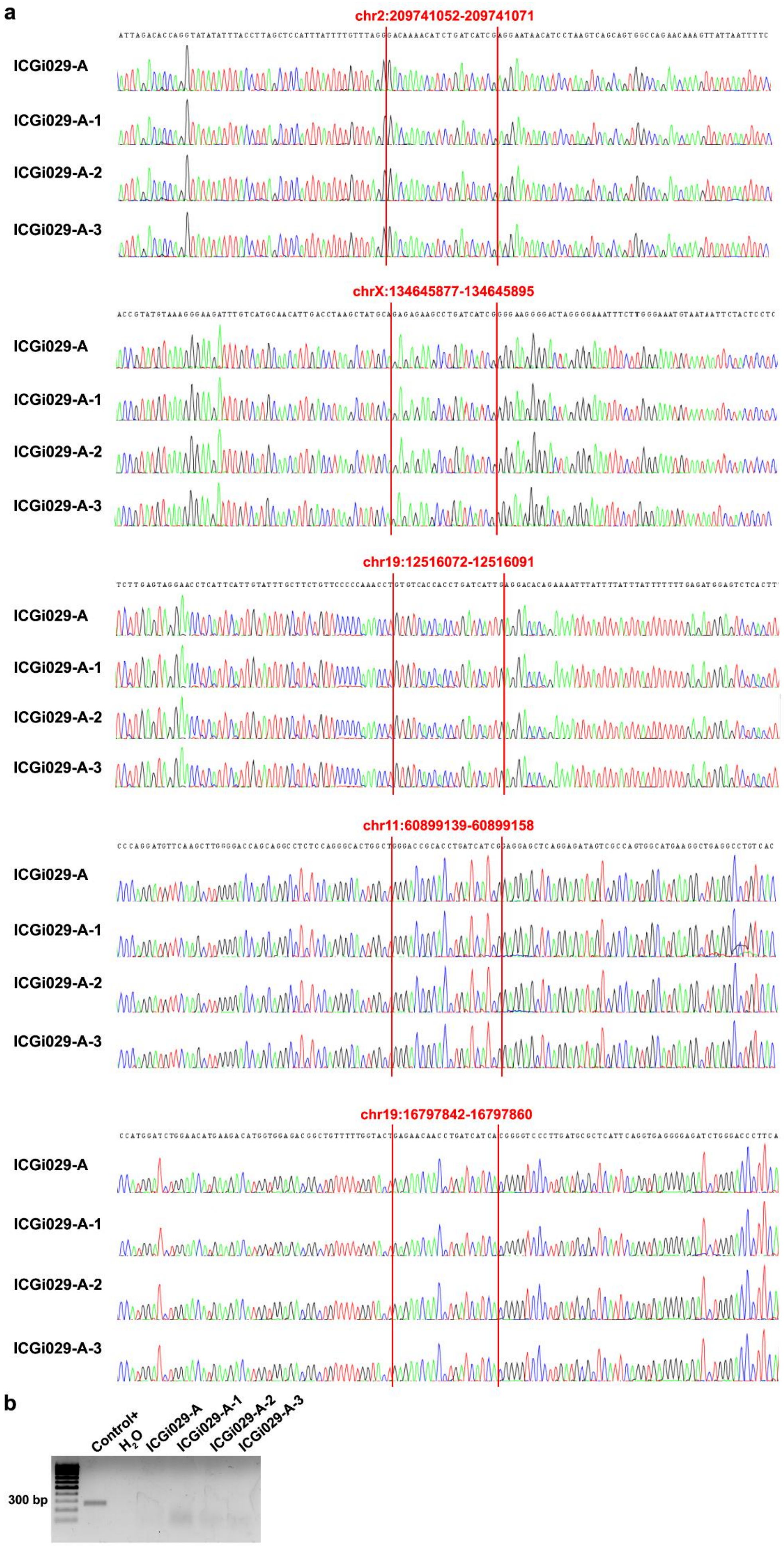
a
chr2:209741052-209741071
ICGi029-A
ICGi029-A-1
ICGi029-A-2
ICGi029-A-3
chrX:134645877-134645895
ICGi029-A
ICGi029-A-1
ICGi029-A-2
ICGi029-A-3
chr19:12516072-12516091
ICGi029-A
ICGi029-A-1
ICGi029-A-2
ICGi029-A-3
chr11:60899139-60899158
ICGi029-A
ICGi029-A-1
ICGi029-A-2
ICGi029-A-3
chr19:16797842-16797860
ICGi029-A
ICGi029-A-1
ICGi029-A-2
ICGi029-A-3
b
Control+
H2O
ICGi029-A
ICGi029-A-1
ICGi029-A-2
ICGi029-A-3
300 bp

Figure S1. Analysis of CRISPR/Cas9 off-target activity and detection of mycoplasma contamination in the iPSC lines with corrected p.N515del (c.1543_1545delAAC) variant in *MYBPC3*. (**a**) Analysis of CRISPR/Cas9 off-target activity in the iPSC lines with the corrected p.N515del (c.1543_1545delAAC) variant in *MYBPC3*. Nucleotide sequences of the CRISPR/Cas9 off-target sites and their surroundings are provided for the patient-specific ICGi029-A iPSC line used for *MYBPC3* editing and three iPSC lines with the corrected p.N515del (c.1543_1545delAAC) variant in *MYBPC3* (ICGi029-A-1, ICGi029-A-2, ICGi029-A-3). CRISPR/Cas9 off-target sites and their positions in the human genome (hg38) are shown in red. (**b**) The iPSC lines with the corrected p.N515del (c.1543_1545delAAC) variant in *MYBPC3* (ICGi029-A-1, ICGi029-A-2, ICGi029-A-3) are not contaminated with mycoplasma. Control+, positive control for mycoplasma contamination. H_2O, negative control. ICGi029-A, patient-specific iPSC line used for *MYBPC3* editing.

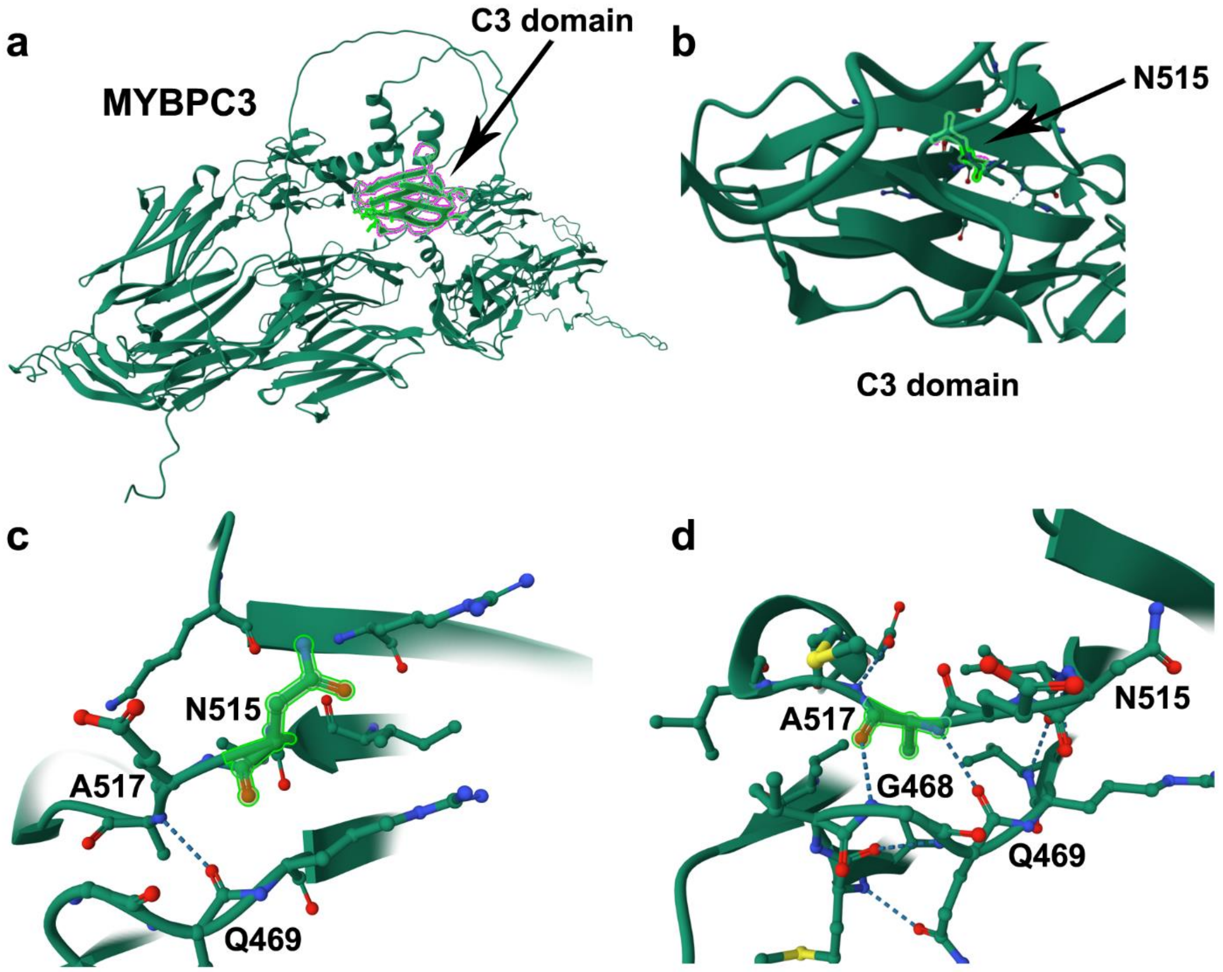

Figure S2. 3D models of MYBPC3, the MYBPC3 C3 domain, and the nearest surroundings of p.N515 generated using RCSB PDB Mol* 3D Viewer (https://www.rcsb.org/3d-view, accessed on 22 November 2024). (**a**) 3D structure of MYBPC3. The C3 domain is highlighted. (**b**) Localization of p.N515 (highlighted in green) in the C3 domain. (**c**) The nearest surroundings of p.N515 (highlighted in green). (**d**) p.A517 forms hydrogen bonds with two other amino acids from the C3 domain, p.Q469 and p.G468. p.A517 is highlighted in green. Hydrogen bonds are shown with blue dotted lines.

Table S1. Oligonucleotides and antibodies used in the study.

	**Oligonucleotides**		
	**Gene/Locus**	**Product Size**	**Nucleotide Sequence (5′-3′)**
Protospacer for CRISPR/Cas9	*MYBPC3*, exon 17	20 b	GAGACACCACCTGATCATCG
Donor oligonucleotide	*MYBPC3*, exon 17	93 b	CGCTAGTGCACAGTGCATAGTGCCCCGCGTCCTCCA GCATGGCTTCGTTGATGATTAGGTGGTGTCTCTGCCC GTCCTTCTTGAACCGGTATT
Analysis of editing events	*MYBPC3*, exon 17	408 bp	CAAATGGTGAGTTCCAGAAGC/ GAGATGAGAAGGATGAGGTTTAGG
Analysis of CRISPR/Cas9 off-target activity	chr2:209741052-209741071	589 bp	TCCTTCATCCCAGGGCATTAT/ ATCTATTCCCTAGAACTGGCATCA
	chrX:134645877-134645895	598 bp	AGGATCCATTGACTCTGATCTCC/ CTGGCCTTGAAATCACCAAAGA
	chr19:12516072-12516091	589 bp	CACCATTGACCCACCCAATTAT/ GCAGGTTGATGGTCAGCTTAAA
	chr11:60899139-60899158	501 bp	GGATGTCAGAGAAAGCCCAGTA/ GAAGCAATTGTTGGCAGTTGTG
	chr19:16797842-16797860	461 bp	CCTCTGGACATGAGAGGTGTAA/ CTCCACTCTGAAGGCACAAATC
Reference gene (RT-qPCR)	*B2M*	90 bp	TAGCTGTGCTCGCGCTACT/ TCTCTGCTGGATGACGTGAG
Pluripotency markers (RT-qPCR)	*NANOG*	116 bp	TTTGTGGGCCTGAAGAAAACT/ AGGGCTGTCCTGAATAAGCAG
	SOX2	100 bp	GCTTAGCCTCGTCGATGAAC/ AACCCCAAGATGCACAACTC
Mycoplasma detection	16S ribosomal RNA gene	280 bp	GGGAGCAAACAGGATTAGATACCCT/ TGCACCATCTGTCACTCTGTTAACCTC
	**Antibodies**		
	**Antibody**	**Dilution**	**Company, Cat #, and RRID**
Pluripotency markers	Mouse IgG2b anti-OCT3/4	1:50	Santa Cruz Biotechnology, Dallas, TX, USA, Cat # sc-5279, RRID:AB_628051
	Rabbit IgG anti-SOX2	1:200	Cell Signaling Technology, Danvers, MA, USA, Cat # 3579, RRID:AB_2195767
	Mouse IgG3 anti-SSEA4	1:200	Abcam, Cambridge, UK, Cat # ab16287, RRID: AB_778073
Markers of differentiated derivatives	Mouse IgG2a anti-TUBB3	1:500	BioLegend, San Diego, CA, USA, Cat # 801201, RRID:AB_2313773
	Mouse IgG2a anti-αSMA	1:100	Dako, Glostrup, Denmark, Cat # M0851, RRID:AB_2223500
	Mouse IgG1 anti-CK18	1:100	Abcam, Cambridge, UK, Cat # ab668, RRID:AB_305647
Cardiomyocyte markers	Mouse IgG1 anti-alpha-actinin-2	1:200	Abcam, Cambridge, UK, Cat # ab9465, RRID:AB_307264
Secondary antibodies	Goat anti-Mouse IgG (H + L) Secondary Antibody, Alexa Fluor 568	1:400	Thermo Fisher Scientific, Waltham, MA, USA, Cat # A11031, RRID:AB_144696
	Goat anti-Rabbit IgG (H + L) Highly Cross-Adsorbed Secondary Antibody, Alexa Fluor 488	1:400	Thermo Fisher Scientific, Waltham, MA, USA, Cat # A11008, RRID:AB_143165

	Goat anti-Mouse IgG3 Cross-Adsorbed Secondary Antibody, Alexa Fluor 488	1:400	Thermo Fisher Scientific, Waltham, MA, USA, Cat # A21151, RRID: AB_2535784
	Goat anti-Mouse IgG1 Cross-Adsorbed Secondary Antibody, Alexa Fluor™ 488	1:400	Thermo Fisher Scientific, Waltham, MA, USA, Cat # A21121, RRID:AB_2535764
